# Preparing Facilitators From Community-Based Organizations for Evidence-Based Intervention Training in Second Life

**DOI:** 10.2196/jmir.3606

**Published:** 2014-09-30

**Authors:** Angel Felix Valladares, Michelle Aebersold, Dana Tschannen, Antonia Maria Villarruel

**Affiliations:** ^1^University of MichiganSchool of NursingAnn Arbor, MIUnited States

**Keywords:** evidence-based intervention, virtual environment, Second Life, health education, community-based organization, facilitator training, computer simulation, distance education, serious gaming

## Abstract

**Background:**

A major barrier to the use and scale-up of evidence-based interventions are challenges related to training and capacity building. A cost-effective and highly interactive multi-user virtual environment, Second Life (SL) is a promising alternative for comprehensive face-to-face facilitator training.

**Objective:**

The purpose of this study was to examine the feasibility of using SL to train facilitators from community-based organizations to use ¡Cuídate! (Take Care of Yourself), one of the few evidence-based interventions developed and tested with Latino youth to reduce sexual risk behaviors.

**Methods:**

We recruited 35 participants from community-based organizations throughout the United States to participate in the SL ¡Cuídate! Training of Facilitators. Preparation to use SL consisted of four phases: (1) recruitment and computer capacity screening, (2) enrollment, (3) orientation to the SL program, and (4) technical support throughout the synchronous training sessions. Technical difficulties, the associated cause, and the mitigation strategy implemented were recorded during each session. Participants completed evaluations including perceptions of self-efficacy and confidence to complete the necessary skills to participate in SL training.

**Results:**

Overall, participants reported high levels of self-efficacy for all skills necessary to participate in SL training. Based on an 11-point scale (0-10), self-efficacy to download and access the software was rated the highest: mean 8.29 (SD 2.19). Interacting with items in SL had the lowest mean score: mean 7.49 (SD 2.89). The majority of technical difficulties experienced by participants were related to inadequate Internet connections or computer malfunctions.

**Conclusions:**

Our findings support the feasibility of using SL for the ¡Cuídate! Training of Facilitators. The process used in this study to prepare participants to use SL can be used as a basis for other evidence-based intervention training in SL. This study is an important contribution to developing cost-effective and accessible training options for evidence-based interventions.

## Introduction

### Background

Multi-user virtual environments (MUVEs), such as Second Life (SL), are a promising alternative to face-to-face training for health care program delivery [1]. While rigorously tested evidence-based interventions (EBIs) have the potential to influence health outcomes, challenges related to training and capacity building access (eg, funding, high travel costs, limited to no technical assistance offered, and limited guidance on appropriate adaptations) [2,3] can impede implementation fidelity and the effectiveness of the EBI. Due to these challenges, more accessible training modalities on how to deliver the intervention accurately are needed to better disseminate EBIs and to ensure their effectiveness. Virtual environments such as SL have not been extensively used for EBI training of individuals who work in community-based organizations and deliver training to adolescents (ie, facilitators). This is important as facilitators deliver the curriculum to the adolescents and facilitate group discussions and other activities that are part of the curriculum. As a cost-effective and highly interactive MUVE, SL has potential to serve as a delivery platform for comprehensive EBI facilitator training.

A MUVE is a computer-based, simulated virtual environment allowing users to inhabit an online virtual world and interact with others via self-representations known as avatars in synchronous sessions [1]. The advantage of MUVEs like SL is that they increase access to capacity building opportunities [2] and are a promising alternative to face-to-face training due to substantial features mirroring human interactions. These features include advanced and realistic voice chat, speech gestures, and the ability to manipulate voices in a multidimensional space just as one would experience in real life [4]. This study describes the process of preparing individuals from community-based organizations to participate in a SL ¡Cuídate! (Take care of yourself) Training of Facilitators [5].

### Second Life Training

SL has been previously used for small-scale health-related programs focusing on education and awareness, support, training, marketing, and promotion of health services [1,6-11]. For example, SL was used to implement a 1-hour seminar to enhance clinician knowledge of insulin therapy for type 2 diabetics for primary care physicians (n=14) [9]. Results of the training indicated a positive impact on self-efficacy to perform insulin therapy, gains in clinical competence around the use of insulin therapy, and positive feedback on the sense of presence attributed to the use of avatars. There was high variability in the SL user learning curve depending on past experience with video games, but an average of 78 minutes was spent with each participant to gain proficiency in SL. Similarly, Mitchell et al pilot-tested motivational interviewing training in SL with primary care physicians (n=13) focusing on colorectal cancer screening [10]. A self-directed approach to learning SL skills was used to reduce the amount of time necessary to prepare participants in SL. The actual amount of time participants spent on preparation was not explicitly reported. Finally, Tschannen et al used SL for diabetes self-management training for nurses. Participants reported that they were highly satisfied with the virtual simulation experience. Further, participants’ ability to apply knowledge gained was comparable to those who completed a face-to-face training alternative [11]. While these studies demonstrate the utility of SL for training, none of these studies reported quantitative data on SL proficiency outcomes, or self-efficacy of SL skills. Further, training participants were limited to highly trained clinicians.

### Technical Challenges

Virtual training presents a number of technical challenges, including computer system capacity and security issues [10]. For example, training participation requires minimum system specifications, installation of a separate computer program, and the development of specific computer skills to facilitate the interaction required in SL to engage fully in the training. Specifically, SL requires a high level of processing power, graphical memory, and a need for a large bandwidth with high-speed Internet access [12]. Individuals may encounter network policies that do not allow access to public virtual worlds like SL or allow downloads of software or a continuous Internet connection [9]. Despite the technological complexities, SL has proven to be an accessible medium for effective training and education.

### Purpose

The purpose of this study was to examine the feasibility of using SL to train facilitators to use an EBI, ¡Cuídate! (Take Care of Yourself), one of the few sexual risk reduction interventions developed and tested with Latino youth [13]. The participants received training in SL to prepare them to deliver the ¡Cuídate! curriculum to Latino youths in their community. This paper examines the management of technical issues and participant self-evaluation of SL skills.

## Methods

### Overview

This is a descriptive study. The study protocol was reviewed by the institutional review board at the University of Michigan and was deemed exempt and not regulated.

**Figure 1:**
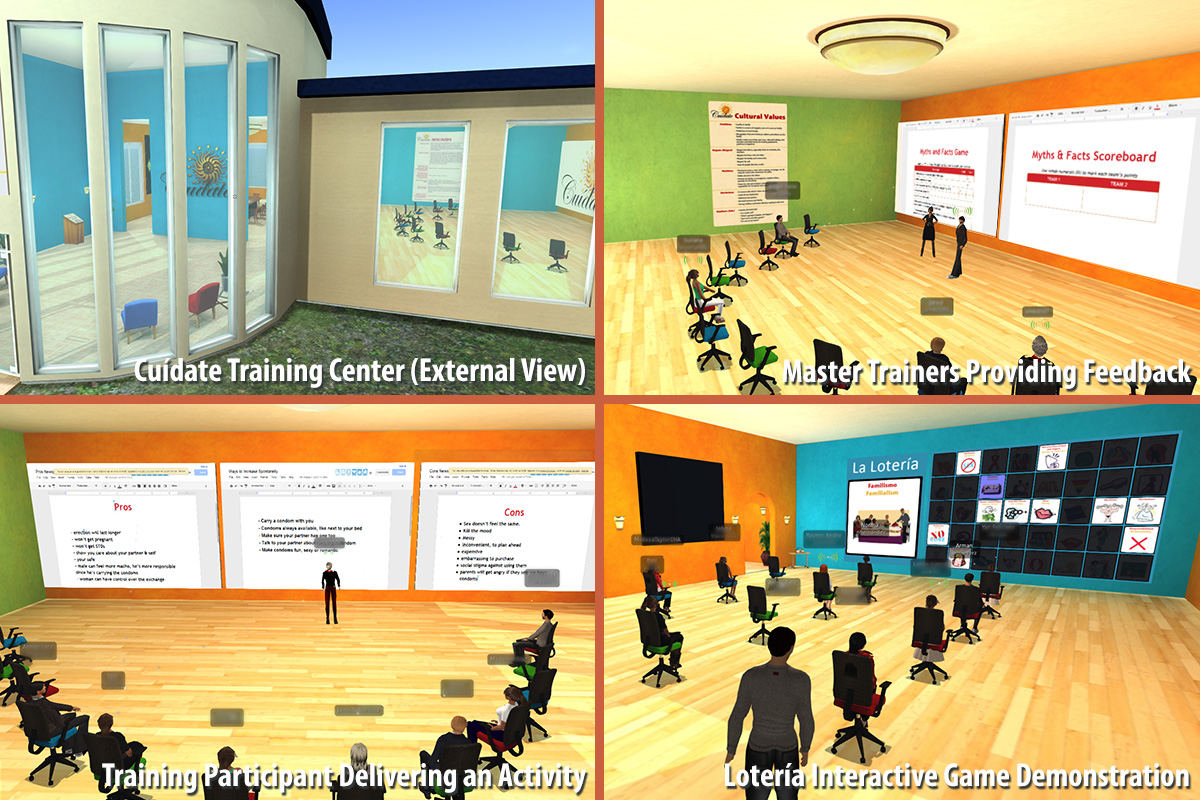
Screenshot of the ¡Cuídate! training center in Second Life.

A virtual training center (Figure 1) was developed in SL to deliver the ¡Cuídate! training. This training center was built on university-owned space in SL (Wolverine Region). The training center consisted of a conference room with a table and chairs and a training room with movable chairs, which were scripted to provide avatars with gestures such as hand-raising. The training was set up with Web prims to display interactive posters and Google Docs.

Preparation to use SL consisted of four phases: (1) recruitment and computer capacity screening, (2) enrollment, (3) orientation to the SL program, and (4) technical support throughout the synchronous training sessions.

### Phase One: Recruitment and Computer Capacity Screening

Community-based organizations throughout the United States were sent information about the SL Cuídate Training of Facilitators opportunity via social media (eg, Facebook, Twitter), distribution lists, and newsletters. To ensure agency staff could feasibly participate in the SL training, an online portal and a screening process were established. Community-based organizations submitted contact information and answered screening questions to determine if their computer system capacity met SL’s system requirements. The minimum system requirements for SL include at least a Windows XP or Mac OS X 10.6 operating system, a graphics card model NVIDIA GeForce 6600 or better, an ATI Radeon 9500 or better, or an Intel 945 chipset for three-dimensional functionality, and a high-speed Internet connection [12]. To identify possible SL installation barriers, access to the Internet (ie, wired or wireless connection) and availability of information technology (IT) personnel at their facility to assist in mitigating technical difficulties was assessed.

If registrants had questionable capacity (eg, older operating systems or a low-power graphics card), research staff guided each registrant through an online scanner available from System Requirements Lab, Inc. [14]. If minimum requirements were met, instructions for downloading and installing the program were provided. In rare cases, a member of the study team walked through the installation steps over the phone with them or their IT personnel.

### Phase Two: Enrollment

Once an agency met the minimum computer system capacity, they were presented with a Memorandum of Understanding (MOU) that included information on the purpose of the study, responsibilities of participants, and expectations and accountability of the research team. Participants received an enrollment phone call to review the MOU and were provided with an outline of the project activities. Once the MOU was signed, a ¡Cuídate! Implementation Kit [15] and a universal serial bus (USB) microphone headset were mailed to participants. The USB microphone headsets were sent to standardize equipment for easier troubleshooting of SL voice chat difficulties during training. For their participation, community-based organizations were provided a financial incentive of US $500.00 to deliver ¡Cuídate! in their community post-participation in the Training of Facilitators.

**Figure 2:**
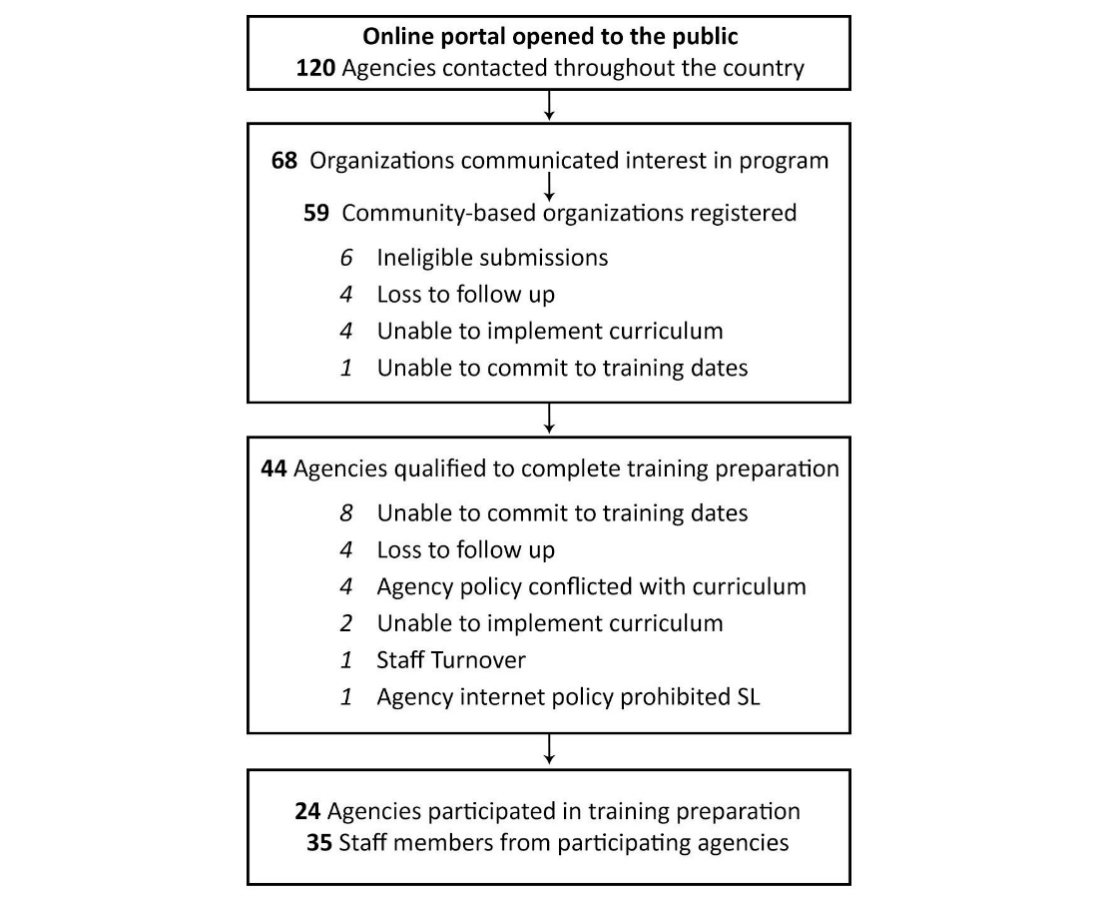
Training participant recruitment and screening process.

Communication occurred with 120 different community agencies and entities throughout the United States via recruitment phone calls. Of those, personnel from 59 agencies underwent the screening process previously described. Of the agencies that met computer system capacity, 55% (24/44) committed to the training and completed the tasks required to participate. Details of the recruitment and screening process are reported in Figure 2.

### Phase Three: Orientation to Second Life

Participants were oriented to SL in three steps: (1) self-directed online orientation manual, (2) a one-on-one SL review session with research staff, and (3) a group skill review on SL training day one. In the first step, we developed an online orientation manual that provided instructions on how to install SL, create an avatar, perform basic navigation functions, teleport to different parts of the environment, manipulate camera angles, and use text and voice communication features. The online manual consisted of text and screenshots of steps and guided video demonstrations embedded within the manual.

In the next step, one-on-one meetings in SL were scheduled to review and practice basic skills and to introduce participants to more complex skills. These additional skills focused on interactions with three-dimensional objects in the program including seats, working on collaborative Google documents [16], changing their display, and participating in small group voice chats. The SL ¡Cuídate! training was developed to limit participant burden with learning SL to ensure the focus of the training was on learning the ¡Cuídate! Facilitator’s Curriculum.

The third step was incorporated into the first day of the SL facilitator training. This orientation session served as an introduction to the ¡Cuídate! training and provided an opportunity to practice features of the SL environment relevant to training. Best practices in SL were shared to ensure a smooth training experience. The practices included silencing microphones when they were not speaking to minimize distracting noises, ensuring their avatars remained active by participating in discussions, raising their avatar’s hand when they had a question or comment, and how best to view the interactive documents and posters during the training.

### Phase Four: Technical Support During Training

In the training center, a designated staff member with high SL proficiency was available to provide technical support. Participants were asked to sign in 15 minutes prior to scheduled SL ¡Cuídate! training and to send a message using text chat to the technical staff if they were having trouble during the session. The staff member would either work in the background with the participant or have them teleport (a quick navigation method in SL) to another location to troubleshoot issues, thus minimizing distractions for other participants.

### Measures

#### Overview

To evaluate the process of preparing individuals to participate in SL training, several different descriptive methods were used. Data were gathered from the participant screening survey, technical support sessions, and a post-orientation survey. The surveys were administered using Qualtrics. Data were then categorized in three different areas for analysis: computer system capacity and experience, SL self-efficacy, and participant preparation time.

#### Computer System Capacity and Experience

A measure of overall computer system capacity consisted of two components: technical specifications and participant experience with online environments. During enrollment, participants were asked about technical specifications related to their operating system and Internet connectivity. Participants were also asked about previous online learning experiences (Yes/No) and if they had used SL prior to this training (Yes/No). They were also asked to report the frequency of engagement in online and gaming software on a 3-point scale (1=rarely, 2=often, 3=very often).

#### Second Life Self-Efficacy

Self-efficacy to use SL was assessed with nine questions adapted from a scale developed by Gist, Schwoerer, and Rosen, which was developed to measure computer and software self-efficacy [17]. The first three questions (eg, User Capability to Navigate SL) measured the participant’s ability to navigate SL after using either the written instructions or the video demonstrations (both part of the orientation manual). The next six questions (eg, Self-Efficacy of SL Skills) in the scale measured the self-efficacy of participants to (1) download SL software, (2) create an avatar, (3) walk with their avatar, (4) teleport to different locations, (5) change the view in SL, and (6) interact with items in SL. The questions were on an 11-point scale, from 0 (completely unable to do the task) to 10 (totally capable of doing the task). Reliability coefficient alpha=.83 for the User Capability to Navigate SL (3 questions) and .87 for the Self-Efficacy of SL Skills (6 questions).

#### Participant Preparation Time

Two questions assessed participants’ estimates of the time spent reviewing the SL orientation manual. Additionally, the research team recorded actual time spent supporting participants in the use of SL or addressing computer issues. Technical difficulties experienced by participants, the associated cause, and the mitigation strategy implemented were also recorded throughout the training and preparation.

## Results

### Overview

A total of 35 participants from 24 agencies participated in 1 of 5 cohorts of SL ¡Cuídate! training. The majority of participants were female (69%, 24/35) and ranged from 20-59 years of age. Most participants had received at least a Bachelor’s degree (80%, 28/35). Slightly more than half of the participating community-based organizations had fewer than 50 employees (58.3%, 14/24), while 10 community-based organizations reported having more than 50 employees (41.7%, 10/24).

### Computer System Capacity and Experience

Most participants had Windows Vista or better (74%, 26/35), while only a few reported using Windows XP (14%, 5/35), or Mac OSX 10+ (9%, 3/35). Regarding Internet connections, nearly equal numbers of individuals (40%, 14/35) reported having a wired connection or only a wireless connection (37%, 13/35), while a small percentage of participants (23%, 8/35) had access to both wired and wireless connections.

A majority of participants (77%, 27/35) reported having online learning experience as well as experience with computer and video games, but very few participants (9%, 3/35) had ever used SL. Of those reporting having played computer or video games in the past 12 months (51%, 18/35), 2 participants (11.1%) played very often, 3 (16.7%) often, and 13 (72%) had rarely played.

### Second Life Self-Efficacy

Most individuals rated their confidence levels high (no mean scores were below 7) on the SL self-efficacy scale. Participants reported high capability to navigate SL after reviewing written instructions and additional video clips (mean 7.97, SD 2.19). Participants had high capability scores for skills such as downloading the SL program and creating an avatar (mean 8.29, SD 2.19, and mean 8.03, SD 2.19 respectively), but slightly lower scores for changing the view on their computer screen and interacting with items in SL (mean 7.49, SD 2.89, and mean 7.49, SD 2.92 respectively). See Table 1.

**Table 1:** Evaluation of SL orientation manual and self-efficacy of SL skills (N=35).

SL skills		Mean (SD)^a^
**User capability to navigate SL**		
	Without reviewing SL orientation materials	6.80 (2.69)
	Reviewing only written instructions	7.80 (1.84)
	Reviewing additional video clips	7.97 (2.19)
**Self-efficacy of SL skills**		
	Downloading the SL software	8.29 (2.19)
	Creating my avatar	8.03 (2.08)
	Making my avatar walk	7.91 (2.79)
	Changing the view on my computer screen	7.49 (2.92)
	Teleporting to different locations	8.00 (2.57)
	Interacting with items in SL	7.49 (2.89)

^a^Mean and standard deviation were derived from an 11-point scale (0=unable, 10=totally capable).

### Participant Preparation Time

The majority of participants (91%, 32/35) reviewed both the written instructions as well as the video demonstrations in the SL orientation manual. The amount of time participants spent using the orientation manual ranged from less than 1 hour (43%, 15/35), between 1 and 2 hours (17%, 6/35) or more than 2 hours (40%, 14/35). It is unknown how many more hours were spent using the orientation manual as the scale ended at 2 plus hours.

The one-on-one session in SL required an average of 28.8 (SD 11.8) minutes per participant with a range of 15-60 minutes. Troubleshooting technical issues required an average of 28.4 (SD 16.4) minutes per participant for 74% (26/35) of the participants. The shortest troubleshooting time (2 minutes) was used to correct simple settings issues, and the longest (75 minutes) was used to assist participants with installation difficulties.

Some participants had issues with SL installation due to either agency policies (n=3) or inadequate skill (n=3). Some agencies had policies prohibiting access to public virtual worlds like SL. The most prominent technical difficulty after installation was headset malfunction and incompatibility with users’ computers. The majority of these issues were corrected by adjusting settings on the operating system or on the SL program itself. For others, there were SL malfunctions such as lags in performance, periodic interruptions to audio or an ejection from the program. Internet connectivity was thought to be the root cause of these performance issues, particularly those with wireless Internet connections. The overwhelming majority of participants who continued to receive troubleshooting during the training reported wireless Internet connections. Technical difficulties and support are highlighted in Table 2.

**Table 2:** Participant technical support and troubleshooting (N=26).

Technical support issue		Instances^a^, N	Possible causes and mitigation strategy
**Phase 2: Enrollment**			
	Installation barriers	6	3 participants had insufficient knowledge to install SL on their own and 3 due to their agency’s network policies; all required assistance installing the program.
	Second Life installation error	1	Laptop PC could not complete installation; participant used another computer for the training.
**Phase 3: Orientation**			
	Headset functioning/ connectivity	15	Linked to Internet connection quality issues as well as settings compatibility with SL and the PC. Audio settings in the operating system and SL were reset, SL was also restarted.
	Could not connect to SL Server	4	Loss of Internet connectivity or use of incompatible Internet devices (eg, cellular Internet hotspots). Participants were required to access SL at an alternate location.
**Phase 4: Technical Troubleshooting in Training**			
	Headset functioning/connectivity	12	Continued connection issues contributed to additional headset issues in training. Audio settings were adjusted, and some restarted SL to regain audio connection.
	SL user navigation issues	5	Users accidentally interact with different features of the program interrupting their view or changing their location. These issues required technical staff to help them navigate back to the correct view or location.

^a^Instances are not equal to unique individuals and include multiple troubleshooting sessions with participants.

## Discussion

### Principal Findings

The purpose of this study was to describe the process and related challenges of preparing individuals to participate in an EBI facilitator training program adapted for SL. The response to initial recruitment for participants was positive despite training being offered in a very non-traditional format (68/120 agencies indicated interest). Throughout the screening process, a variety of agencies were able to meet technical requirements and complete the training, half of which were small community-based agencies (<50 employees). Reasons that agencies did not participate, despite initial interest, were primarily related to inadequate computer system capacity and/or the inability to implement the training within 6 months post training. The variation in computer system capacity and Internet connections of the agencies that participated allowed for the opportunity to test the feasibility of using SL in areas that do not have available technical support staff or high-speed Internet capabilities.

In terms of SL skills orientation, the majority of participants reported high confidence levels for basic skills in SL. This supports the feasibility of using a self-directed orientation manual since participants were able to reasonably orient themselves to the SL environment without additional support. Further, the use of a self-directed orientation manual resulted in limited staff time (less than one hour) for SL orientation and limited time necessary for additional troubleshooting. The time spent on orientation is less than previous reported results of Wiecha et al who spent 78 minutes coaching each participant [9]. Findings from this study provide the overall process that can be implemented to facilitate other similar trainings in SL.

### Lessons Learned

In this study, participants did identify technical challenges that impacted facilitator training. Major difficulties encountered included hardware and software compatibility issues and Internet issues (bandwidth) and not with individual ability to use the SL software (ratings for self-efficacy were high and very few troubleshooting encounters were reported requiring assistance with SL program skills). Although system requirements were identified pre-training, further testing is needed to determine specifically what system requirements should be mandated in order to prevent technical hurdles. For some, the use of a laptop interfered with a seamless experience because some laptops are not designed with the capacity (ie, adequate graphic cards, reliance on wireless) to run even less burdensome three-dimensional environments such as SL. Of these connection-related difficulties, audio input and output malfunctions were most prevalent even when using a standard audio headset and after pre-training audio testing.

One unanticipated cause of these issues was the load on an individual’s network and computer system when multiple users were simultaneously logged on to the virtual environment. Smaller training cohorts (fewer than 7 participants) had fewer connection-related issues in the live training. Therefore, providing installation instructions upon initial registration may allow registrants to test their bandwidth by having them enter more populated areas of SL to see if their computers can handle a large number of users. This early bandwidth testing should reduce the number of technical difficulties that may arise in the training program from a lack of bandwidth capacity. One additional approach that may be warranted is to limit the amount of participants per training session to minimize the technical challenges that may occur.

### Conclusions

Technical support was a vital component of this study. Delivering training in SL requires technical expertise to remotely troubleshoot with participants. Advanced knowledge of both computer hardware and software components is essential for the technical staff who will provide the support before and during training. By having support staff available throughout the training, major interruptions were avoided and participant burden was minimized allowing for the successful retention of all 35 participants across 3 days of training.

In summary, preparing participants to use SL for the ¡Cuídate! Training of Facilitators was feasible, and participants were confident in their ability to use the program for training after completing orientation. In addition, we developed processes to decrease technical difficulties prior to and during training. The potential advantages of conducting SL trainers include the ability to access EBI training without the high cost of travel. This is especially relevant for agencies and communities with limited resources. Decreasing access barriers through the use of technology can facilitate the dissemination and use of evidence-based interventions, resulting in improved health outcomes.

## Abbreviations

EBI: evidence-based intervention
IT: information technology
MOU: memorandum of understanding
MUVE: multi-user virtual environment
SL: Second Life
USB: universal serial bus
